# Cross-Talk Between Gut Microbiota and Adipose Tissues in Obesity and Related Metabolic Diseases

**DOI:** 10.3389/fendo.2022.908868

**Published:** 2022-07-05

**Authors:** Dan Wu, Huiying Wang, Lijun Xie, Fang Hu

**Affiliations:** National Clinical Research Center for Metabolic Diseases, Key Laboratory of Diabetes Immunology (Central South University), Ministry of Education, Metabolic Syndrome Research Center, and Department of Metabolism and Endocrinology, The Second Xiangya Hospital of Central South University, Changsha, China

**Keywords:** obesity, gut microbiota, adipose tissues, energy metabolism, inflammation

## Abstract

The rapid increase of obesity and associated diseases has become a major global health problem. Adipose tissues are critical for whole-body homeostasis. The gut microbiota has been recognized as a significant environmental factor in the maintenance of energy homeostasis and host immunity. A growing body of evidence suggests that the gut microbiota regulates host metabolism through a close cross-talk with adipose tissues. It modulates energy expenditure and alleviates obesity by promoting energy expenditure, but it also produces specific metabolites and structural components that may act as the central factors in the pathogenesis of inflammation, insulin resistance, and obesity. Understanding the relationship between gut microbiota and adipose tissues may provide potential intervention strategies to treat obesity and associated diseases. In this review, we focus on recent advances in the gut microbiota and its actions on adipose tissues and highlight the joint actions of the gut microbiota and adipose tissue with each other in the regulation of energy metabolism.

## 1 Introduction

Obesity, a chronic disease characterized by the excessive expansion of adipose tissues and associated low-grade inflammation, is the risk factor for various disorders, including insulin resistance (IR), metabolic syndrome, hypertension, type 2 diabetes mellitus (T2D), cardiovascular diseases, nonalcoholic fatty liver disease (NAFLD), cancers and mental diseases (1, 2). Genome-wide association (GWAS) studies have found that fat mass and obesity-associated (FTO) gene and its single nucleotide polymorphisms (SNPs) are associated with obesity (3). Such as SNP rs 1558902 was proved to be associated with BMI in a study including 247,796 Europeans (3). Besides genetic deficiency, such as leptin or leptin receptor deficiency (4, 5), obesity is mainly caused by a disrupted balance of energy homeostasis with increased nutrition intake and decreased energy expenditure (6). New therapeutic strategies through reductions in energy intake, absorption, or storage are crucial to fight against the worldwide epidemic of obesity.

Although obesity is characterized by excessive accumulation of adipose tissues, under physiological conditions, adipose tissues are critical for whole-body homeostasis, including lipid storage, thermoregulation, and secretion of adipokines to regulate energy balance, metabolism, and immune responses (7). According to different phenotypes, distribution, and physiological functions, adipose tissues are classified into white and brown adipose tissues. White adipose tissue (WAT), as the majority form of the adipose tissue mass in our body, is mainly responsible for energy storage in the form of triglycerides (TG) (7). It also secretes many adipokines, such as leptin and adiponectin, to regulate whole-body homeostasis (7). Brown adipose tissue (BAT) represents a small part of the total fat mass and is located predominantly in the interscapular and supraclavicular regions of adult humans (8). BAT expresses unique uncoupling protein 1 (UCP1), which eliminates the difference in transmembrane proton concentration between the two sides of the mitochondrial inner membrane and blocks the adenosine triphosphate (ATP) synthesis, resulting in the dissipation of energy as heat (9). Thus, BAT is involved in thermoregulatory, energy expenditure, and insulin sensitivity. An inducible form of brown-like adipocytes, namely beige adipocytes, can be found interspersed in WAT depots (10). Similar to brown adipocytes, beige adipocytes also express UCP1 and are involved in non-shivering thermogenesis and the dissipation of energy in response to cold exposure or β-adrenergic receptor (βAR) activators (10, 11). WAT, BAT, and beige adipocytes are important for energy balance, and their dysfunctions are closely associated with the pathogenesis of obesity and metabolic diseases.

Recently, gut microbiota has been viewed as an important regulator for the maintenance of host energy homeostasis and immunity (12). In human digestive system, there are around 10 trillion microbiota, carrying more than 9.9 million microbial genes (12, 13). Gut microbiota has lots of functions, including keeping in charge of metabolism and nutrition absorption, protection of the integrity of intestinal mucous, and regulation of the immune responses. In 2004, Gordon and colleagues first verified that gut microbiota is a crucial environmental factor that regulates energy homeostasis and fat accumulation (14). After that, with the development of sequencing techniques and bioinformatics (13), lots of studies have demonstrated a close relationship between gut microbiota and the host energy homeostasis. Studies in both mice (15) and humans (16) have shown that obesity is closely linked to the altered gut microbiota, including lower microbial richness and diversity and decreased levels of *Bacteroidetes*, one of the most abundant phyla in the gut (17, 18). Altered microbial composition is related to the alterations in the gut microbial metagenome, especially an enrichment of genes involved in energy harvest (14). Nutrients/different diets, medication, and basic metabolic state (energy balance, etc.) of the host can impact the abundance or composition of the gut microbial population, which, in turn, may affect not only the host’s energy expenditure (weight loss) but also energy storage (weight gain) (19).

A growing body of evidence suggests that the gut microbiota is a bridge to connect the external environment and the host homeostasis. On the one hand, the composition of gut microbiota can be affected by region (20), race/ethnicity (21), diet (22–24), and host genetics (6). For example, geographical location has been suggested to be closely associated with gut microbiota variation (20), and race/ethnicity (22) or its correlates such as diet and socioeconomic status have important influences on gut microbiota. Western diet with high fat and low fiber is one of the most significant drivers of metabolic diseases (21, 25). Besides, host genetics can have a fundamental influence on the composition and function of gut microbiota. Interactions between host genetics and diet modulate gut microbiota and susceptibility to obesity and metabolic diseases (25, 26).

On the other hand, gut microbiota modulates host homeostasis by the distal organs such as liver (27) and adipose tissues (28) *via* metabolites and cytokines. The liver communicates with the intestine by bile acids (BAs) and many bioactive mediators such as hepatokine FGF21 (27). Gut microbiota regulates energy expenditure and alleviates obesity by promoting browning of WAT, the activity of BAT, and lipid metabolism. Gut microbiota also produces specific microbial-derived metabolites and structural components, such as short-chain fatty acids (SCFA), lipopolysaccharide (LPS), and peptidoglycans, which may act as the central factors in the pathogenesis of inflammation, insulin resistance, and obesity (18, 29). Thus, understanding the relationship between gut microbiota and adipose tissues and the potential mechanism may provide new approaches to treat obesity and related metabolic diseases. In this review, we will focus on recent advances in the connection between gut microbiota and adipose tissues and highlight the mutual actions of gut microbiota and adipose tissue with each other in the regulation of energy metabolism.

## 2 Gut Microbiota and Adipose Tissues - Two Edges of One Sword

A growing body of evidence suggests that the relationship between gut microbiota and adipose tissue is complicated. It may influence the development of metabolic alterations of white adipose tissues associated with inflammation or the activity of brown and beige adipose tissues for energy expenditure and weight loss.

### 2.1 Gut Microbiota Regulates Adipose Tissue Expansion and Lipid Metabolism

Earlier studies have shown that gut microbiota participates in the development of adipose tissues in both normal and pathological conditions. Gut microbiota’s ability to harvest energy has been proposed as a major contributor of conventional mice (harbored a microbiota beginning at birth) in fat storage. Conventional mice have 42% more total body fat mass than germ-free (GF) mice (raised in the absence of any detectable microorganisms) (14). Adult GF mice transplanted with the gut microbiota, which is harvested from the distal intestine (cecum) of conventionally raised mice, produce a 60% increase in body fat content within 2 weeks (14). Obesity-associated gut microbiome can increase the capacity for energy harvest from the diet, thus contributing to increased adipose tissue storage of the host, especially the WAT (30). Transplantation of gut microbiota isolated from obese mice to GF mice resulted in a greater increase in total body weight and fat mass compared with colonization with gut microbiota from lean donors (30). And GF mice have a significant reduction of energy harvest that is accompanied with thinner intestinal villi, larger cecum, and decreased inflammatory responses (30, 31). Mechanistically, GF animals are protected from diet-induced obesity by inducing fatty acid metabolism through elevated levels of fasting-induced adipocyte factor (Fiaf)/angiopoietin-like protein 4 (Angptl4). Angptl14 is a circulating lipoprotein lipase (LPL) inhibitor, which can be selectively suppressed in the gut epithelium by the microbiota, and/or through increased AMP-activated protein kinase (AMPK) activity (14, 32).

The dynamic linkage between adiposity and gut microbial ecology has also been observed in human subjects. People with obesity tend to have higher levels of the phylum *Firmicutes* and fewer *Bacteroidetes* counts than normal-weight individuals (33). A study including more than 800 individuals indicates that gut microbiota may explain 2.74% variation in BMI, 2.46% of HDL in plasma, and 3.8% of TG in plasma (34). A crossover clinical trial with 21 individuals observed that increases of 20% in *Firmicutes* and decreases of a corresponding in *Bacteroidetes* were linked to an increased energy harvest (35). Similar phenomena were observed in obese children (36) and adult female twin pairs discordant for obesity (37). Using fecal microbiota transplantation from one in twin pairs to mouse respectively, researchers found that the increased fat mass of mice who had received an obese twin’s fecal microbiota was notably higher than the mice that received a lean twin’s fecal microbiota (37).

Omics analysis revealed significant differences between conventional and GF mice in their global adipose lipid (38, 39). The gut microbiota significantly increases triglyceride (TG) levels in the WAT, which is correlated with increased adipose tissue mass in conventional mice, compared with GF counterparts. Interestingly, TG levels were also higher in the liver of conventional mice, but lower in their serum, indicating increased lipid clearance by microbiota (39). Roy et al. found that compared with GF mice, conventional Apoe^-/-^mice had higher plasma cholesterol levels, hepatic cholesterol, and HDL cholesterol levels. Moreover, transplant of gut microbiota from humans elevated plasma cholesterol levels in mice, which might correlate with *Betaproteobacteria, Alistipes*, *Bacteroides*, and *Barnesiella tax* (40). These lipidomic results are in agreement with previous discoveries showing that microbiota increases LPL and promotes lipid clearance through suppression of Angptl4/Fiaf expression (32). LPL helps TG enter the circulation system through the liver and then TG is absorbed and stored by fat cells (41).

Certain strains of bacteria play an important role in nutrient absorption. Tazi et.al, using two models of commensal microorganisms, *Lactobacillus paracasei* and *Escherichia coli*, demonstrated the role of gut microbiota in regulating lipid absorption and metabolism through the epithelium. It has been reported that under homeostatic conditions, *L. paracasei* boosts lipid accumulation in enterocytes, while *E. coli* raises lipid catabolism and suppresses chylomicron circulating levels through the inhibition of mTOR pathways (42). This is consistent with the results in human studies (16, 35). However, *L. paracasei*-colonized mice but not *E. coli-*colonized mice resist high-fat-diet (HFD)-induced hypercholesterolemia and an increase in body weight (43). Conversely, Aronsson et al. demonstrated that the probiotic bacteria *L. paracasei ssp paracasei F19* (F19) decreases lipid accumulation in mice fed with HFD by increasing Angptl4 and AMPK activity (32, 44). These two contradictory observations may be a result of the complex interplay between host genetic factors, environmental factors, or the biological properties of gut microbiota.

Although currently available data suggest that gut microbiota is closely associated with increased fat mass by promoting lipid absorption, lipogenesis, and adipogenesis, the underlying molecular mechanisms are still unclear and deserve further investigation.

### 2.2 Gut Microbiota Promotes Browning of WAT and Activity of BAT

#### 2.2.1 Cold Exposure and Microbiota Depletion

It has been reported that beige adipocytes can be developed from white adipocytes through a process known as browning. Various stimulus, including cold temperature, β3-AR agonists, and circulating hormones, such as fibroblast growth factor 21(FGF21), can turn white adipocytes into beige adipocytes (11). Several studies have indicated that gut microbiota is a vital endogenous factor that regulates the browning of WAT and activation of BAT to adapt to external environment changes (38, 45)

Mestdagh et al. first showed that gut microbiota modulates the lipid metabolism in BAT. The absence of gut microbiota enhances BAT lipolysis and suppresses lipogenesis in GF mice, suggesting activated lipid catabolism in their BAT (38). In line with this study, another study has demonstrated that microbiota depletion, either by antibiotic treatment or in GF mice, promotes the functional beige adipocytes development in subcutaneous and perigonadal visceral adipose tissues. It increases the expression of brown fat markers, such as PPAR-γ coactivator 1a (Ppargc1a), Ucp1, and acyl-CoA synthetase long-chain family member 1 (Acsl1), and improves glucose tolerance and insulin sensitivity, while decreasing white adipose tissue mass in both genetically and diet-induced obese mice (46, 47). It has been proposed that eosinophil infiltration and enhancements of type 2 cytokine signaling and M2 macrophage polarization in the WAT, which are known to drive the process of browning, may contribute to the above metabolic improvement of the mice (46).

Previous research has indicated that cold exposure can notably change the gut microbiota composition by decreasing the richness of most gut microbiota phylum, especially *Bacteroidetes, Proteobacteria, Tenericutes, Actinobacteria, Verrucomicrobia*, and *Cyanobacteria*, while increasing *Firmicutes, Deferribacteres*, and *Firmicutes* versus *Bacteroidetes ratios* (47–49). Transplantation of the “cold microbiota” to GF mice can increases insulin sensitivity, cold tolerance as well as fat loss in the host. The expression of thermogenic genes is markedly elevated, especially Ucp1, Prdm16, Pgc-1α, and PPARγ in the BAT, as well as in the subcutaneous WAT (47), indicating WAT browning effects caused by “cold microbiota” transferring. In line with this, Zietak et al. observed a notable change in the gut microbiota composition at the family and phylum levels upon acute and long-term cold exposure. There were increased levels of *Mogibacteriaceae, Ruminococcaceae, Adlercreutzia*, and *Desulfovibrio* and reduced levels of *Erysipelotrichaceae, Bacilli*, and the genus rc4-4. Respectively, these genera are linked to leanness and obesity (49). GF mice that were fed a HFD at room temperature gained improved glucose tolerance and less adipose tissue, which is associated with increased BAT thermogenesis and a plasma bile acid profile (49). When rats were exposed to cold for 4h/day for 21 days, both subpopulations of *Clostridiale* and concentrations of butyric and isovaleric acid increased whereas *Bifidobacteria* decreased in the caecum (45). The *Clostridiale* subpopulation is the main producer of butyrate, particularly in *Clostridial* clusters IV and XIVa (45). These studies have further suggested that reducing ambient temperature can fight obesity by altering gut microbiota composition and promoting thermogenesis.

Cold exposure not only alters the gut microbiome, but also modulates bile acid synthesis pathway by up-regulating the expression of crucial enzyme cytochrome P450, polypeptide 1 (CYP7B1), family 7, and subfamily b in the liver. This sets off a metabolic process that integrates lipoprotein clarification in BAT and hepatic conversion of cholesterol to bile acids synthesis pathway, leading to increased hepatic synthesis and more fecal excretion of bile acids, which distinctly changes the gut microbiota composition and increases heat production (50). This study has identified a novel BAT-liver-gut axis in the regulation of energy metabolism. Furthermore, in a study of 12 healthy female subjects, oral supplementation of the chenodeoxycholic acid (CDCA) could increase BAT activity and whole-body energy expenditure (51).

AMPK, an essential nutrient sensor for maintaining cellular energy status, plays important role in regulating glucose metabolism in various tissues. Very recently, Huang et al. identified that intestinal AMPKα1 stimulates thermogenesis by modulating anti-microbial peptide that manipulates gut microbiota and metabolites. These intestine-BAT communications through AMPKα- related signaling might partially be the underlying mechanism of beneficiary action of metformin on the intestine (52).

However, a recent study by Li et al. has reported different results by showing that microbiota depletion *via* antibiotic treatment or in GF mice impairs the browning of WAT (53). They have reported that the Ucp1-dependent thermogenesis is blunted with the depletion of gut microbiota when exposed to cold stress, which effect can be partly rescued by butyrate indicating that this metabolite may play an important role in the normal thermogenic responses to cold temperature. The study has also shown that alternative macrophages, eosinophil, and type 2 cytokine signaling have no influence on thermogenesis and energy expenditure in GF mice (53, 54). The opposed results among different studies may be related to different backgrounds of gut microbiota compositions caused by different housing conditions, dietary nutrients, sex and age of rodents, antibiotic administration methods (drinking water or gavage), or other unknown factors that deserve further investigation in the future.

Although recent studies suggest that the gut microbiota is the key player in the regulation of energy balance and glucose homeostasis *via* thermogenesis, several studies have also shown that the ability of gut microbiota the maintenance of glucose homeostasis is independent of the manipulation of energy expenditure. By using commensal depleted (CD) and GF mice models, Krisko and colleagues have demonstrated that the gut microbiota seems disposable for both cold- and diet-induced thermogenesis and not required for the recruitment and activation of thermogenic tissues. The gut microbiota maintains blood glucose by regulating hepatic gluconeogenesis rather than adipose tissue in cold (55). However, in the absence of changes in energy expenditure, the gut microbiota seems to promote hepatic gluconeogenesis to maintain glucose homeostasis through the production of amino acid metabolites to facilitate hepatic tricarboxylic acid (TCA) cycle fluxes for gluconeogenesis (55), whose effect is independent of adaptive thermogenesis. A very recent study has found that gut microbiota depletion through antibiotic cocktail treatment promotes glucose uptake and clearance in BAT and cecum, whose effect is dissociated from adaptive thermogenesis (28).

Taken together, current studies have demonstrated that cold temperature and microbiome deletion or transplantation can decrease adiposity through the induction of WAT browning and BAT activity. Further studies on the mechanisms underlying these actions are needed to explore the relationship between gut microbiota and browning of WAT and activity of BAT.

#### 2.2.2 Intermittent Fasting and Caloric Restriction

Intermittent fasting (IF) is known as an effective and useful strategy for weight loss and has multiple benefits for metabolism and health (56). Recent studies have shown that IF can induce WAT browning by altering gut microbiota composition. Li et al. reported that an every-other-day fasting (EODF) stimulates the browning of WAT, and greatly ameliorates obesity and associated hepatic steatosis and insulin resistance (57). These effects are linked to a change of the gut microbiota composition by increasing the Operational Taxonomic Unit (OTU) abundance of *Firmicutes* while decreasing most other phyla and, as a consequence, elevating serum levels of lactate and acetate (57). Both lactate and acetate have been reported to induce WAT browning, *via* the proton-linked monocarboxylate transporter 1 (MCT1) in WAT and BAT (58, 59). Consistently, transplantation of microbiota from IF mice to GF mice significantly upregulates inguinal WAT Ucp1 expression and increases small intestine length (57). Kim et al. thought IF promoted adipose thermogenesis *via* vascular endothelia growth factor (VEGF) expression in WAT and the expression of Adrb3, Cidea and Ucp1 were significantly elevated in HFD-IF mice, which might due to β-AR-dependent thermogenesis (60).

Similar to IF, caloric restriction (CR) also induces metabolic improvements and stimulates the browning within the subcutaneous and visceral adipose tissues (61). Change of microbiota not only contributes to increased activity of BAT and browning of WAT during CR, but also promotes metabolic improvements, including improved glucose tolerance, insulin sensitivity, and decreased fat gain (62). During CR, the most dramatic changes in the composition of gut microbiome are the increases in *Erysipelotichaceae* and *Lactobacillaceae* and decreases in other Firmicutes families, as well as increased levels of *Bacteroidaceae* and *Verrucomicrobiaceae* (62). In a study with 49 overweight and obese adults, increased *Akkermansia muciniphila* during CR has been shown to reduce adiposity and increase insulin sensitivity (63), which may contribute to the metabolic benefits of CR. Very recently, Li and colleagues discovered that CR could reshape the gut microbiota by elevating the ratio of *Firmicutes* to *Bacteroidetes*. In particular, *Parabacteroides distasonis* was significantly reduced (64). *Parabacteroides distasonis* is able to produce secondary bile acids (BAs) and is known to ameliorate weight regain (64).

In line with those observations from murine studies, data collected from patients also suggests a connection between microbiota and adipose tissue physiology in humans. By analyzing gut microbiota and browning gene markers in WAT from 34 morbidly obese subjects, Moreno et al. have found that, in people with obesity and insulin resistance, relative abundance (RA) of *Firmicutes* decreases, whereas compared with insulin sensitive subjects, *Bacteroidetes* and *Proteobacteria* RA increase (65). Interestingly, *Firmicutes* RA is positively linked to browning markers (Ucp1, Prdm16, and Dio2) in subcutaneous but not in visceral adipose tissue. Specifically, those bacteria belonging to the *Ruminococcaceae* family, are positively connected with plasma level of acetate, which is also associated with increased beige fat development and insulin sensitivity (65). A clinical trial based on 24 patients with metabolic syndrome demonstrated that 10-hour time-restricted eating decreased body weight, waist circumference, blood pressure, LDL cholesterol, hemoglobin A1C, and the risk of cardiovascular disease in 12 weeks (66). Very recently, a random clinical study with 139 patients found that CR and time-restricted eating have similar effects on reducing body weight, body fat, or metabolic risk factors (67). However, pharmacological means of inducing beiging and thermogenesis in humans by environmental (e.g., cold) or nutritional (e.g., IF, CR) manipulation and the roles of microbiota are still unknown and deserve further investigation.

In summary, the gut microbiota plays an essential role in whole-body energy metabolism by regulating thermogenic programs in different fat depots. Some environmental and nutrition situations (e.g., cold stress, fasting, or CR) could induce a shift of gut microbiota composition that stimulates browning of WAT and activity of BAT. However, the underlying mechanisms and pathways have not yet been fully identified.

### 2.3 Gut Microbiota and Inflammation of Adipose Tissues

Chronic low-grade inflammation is a trademark of many metabolic diseases, such as obesity, T2D, and NAFLD (68, 69). During obesity, the expanded adipose tissues develop hypoxia and release pro-inflammatory cytokines and chemokines, leading to the infiltration of immune cells (including macrophages, natural killer cells, neutrophils, and T cells) and secretion of pro-inflammatory factors such as tumor necrosis factor (TNF), interleukin 1beta (IL-1b), and IL-6 (70–72). A growing number of evidence suggests that gut microbiota play essential roles in adipose tissue inflammation in obesity and related disorders.

Changes in the abundance and diversity of microbiota are associated with inflammation in obesity. Using metagenomic approaches, Cotillard et al. demonstrated that people with 40% reduced microbial gene richness have more noticeable dysmetabolism and low-grade inflammation (73). An increase of gene richness and diversity is linked to a significant decrease in adipose tissues, circulating cholesterol, and inflammation. In addition, it is suggested that certain ‘pro-inflammatory’ bacterial strains, such as *Ruminococcus gnavus* or *Bacteroides* species, might prevail and certain ‘anti-inflammatory’ strains, such as *Faecalibacterium prausnitzii*, are less prevalent in obesity (14, 73, 74).

Not only does HFD feeding have profound impacts on gut microbiota composition, but it can also cause anatomical and functional changes of the intestinal barrier. Disruption of the intestinal barrier can cause low-grade inflammation in the small bowel and color locally. And some bacteria products such as LPS could leak into plasma inducing systemic inflammation (75). Studies in animals and humans have demonstrated that circulating LPS levels are one of the key elements linking the gut microbiota and inflammation of adipose tissues. Cani et al. found that HFD increases the fraction of an LPS containing microbiota in the gut accompanied with increased F4/80-positive cells and markers genes of inflammation in WAT. LPSCD14 system participates in insulin resistance and the onset of diabetes and obesity (76).

It has been hypothesized that LPS engages in the inflammatory process in adipose tissue during obesity (77). On the one hand, LPS is involved in the transition of the M2 to the M1 macrophages, the latter which causes inflammation responses of adipose tissues; on the other hand, LPS may activate caspase-4/5/11signaling, which induces pyroptosis, an inflammatory kind of programmed cell death of adipocytes. The dead adipocyte remnants and macrophages compose a morphological inflammation marker called crown-like structure in adipose tissue (78). In addition, LPS can activate receptor TLR4 on adipocytes and stimulate pro-inflammatory pathways. The infiltration of macrophages and the up-regulation of TNF-α, NF-κB, and IL-6in turn promote adipose tissue inflammation (79, 80). Also, the LPS-activated TLR leads to the downstream assembly of a complex of LPS-binding proteins such as CD14, LBP, and myeloid differentiation factor-2 (MD-2), leading to the formation of the activated (TLR4–MD-2–LPS) complex (74, 81). Orret al. demonstrated that global TRL4 deficiency could reduce body fat gain slightly, reduce liver TG and promote M2 polarization of macrophages in adipose tissues after HFD (82). Additionally, it has also been demonstrated that CD36 knockout mice fed with HFD showed reduced adipose tissue inflammation and suppressed pro-inflammatory cytokine response when exposed *ex vivo* to LPS, indicating that CD36 has an important impact on TLR signaling pathway (83). And gut microbiota can also produce some metabolites to reduce LPS-associated inflammation, such as SCFA, polyamines, and aryl hydrocarbon receptor (AHR) ligands (84). In many animal studies, an SCFA butyrate has been thought to act as an anti-inflammation metabolite (83) to block the translocation of LPS in the intestines, thus ameliorating LPS-induced pro-inflammatory effects (85, 86).

Previous studies have shown that probiotic strains can suppress adipose low-grade chronic inflammation by modulating the host’s immune responses (87). Lactic acid bacteria (LAB) strains can suppress chronic inflammation in adipose tissues by regulating the secretion of adipokines (73). *Lactobacillus casei* CRL431 administration decreased inflammatory cytokines, includingIL-6, TNF-α, and MCP-1, in adipocytes and macrophages in diet-induced obesity (74, 88). Additionally, evidence collected from *in vitro* (89) and *in vivo* (90, 91) studies have shown that lactic acid bacteria, especially strains of *Lactobacillus* and *Bifidobacterium* genera present anti-inflammatory properties in adipose tissue (91). The mucin-degrading bacterium *Akkermansia muciniphila* has anti-obesogenic effects in mice and humans (63) and treatment of HFD-fed mice with *A. muciniphila* attenuated visceral WAT inflammation by reducing the levels of pro-inflammatory cytokines, including IL-6 and IL-1β, and increasing the number of regulatory T cells (Tregs) (92). *Faecalibacterium prausnitziiare* is an important butyrate producing bacteria and plays critical roles in gut homeostasis. Xu et al. have demonstrated that, under diabetic condition, *F. prausnitzii* produced anti-inflammatory molecules and restored the intestinal barrier structure *via* increasing the expression of tight conjunction protein ZO-1in humans (93). Oral administration of *F. prausnitzii* markedly reduced the inflammation of colon by blocking NF-κB activation and IL-8 production (94). *F. prausnitzii* may therefore serve as a biomarker to diagnose gut related diseases and metabolic diseases. Due to their potential benefits on health, probiotics have been used in food and health care medicines (95). *Parabacteroides distasonis* has been identified to play a protective role in obesity (96). Xu et al. found that with the treatment of Panax notoginseng saponins (PNS), a traditional Chinese medicine, the obese mice had a higher AR of *P. distasonis* and *A.muciniphila* and lower body weight and fat mass (97).

Interestingly, dietary lipids can also influence adipose tissue inflammation. Unsaturated lipids were reported to decrease body weight gain, increase *Firmicutes* to *Bacteroidetes* ratio and promote a healthier metabolic phenotype, while saturated lipids aggravate WAT inflammation and promote a higher degree of obesity, which is partly attributed to distinct gut microbiota richness and diversity (24, 98). Mechanistically, gut microbial-derived factors increase chemokine CCL2 expression in adipocytes, which buildups macrophage accumulation and inflammation in WAT through TLR4, MyD88, and TRIF related pathways (98). In line with these studies, Tran et al. found that consumption of western-style diet (WSD), a low-fiber and high-fat diet, prompts adiposity and adipose inflammation characterized by increased M1to M2 macrophage ratio and proinflammatory cytokine expression. Microbiota ablation suppresses macrophage infiltration and M1 macrophage polarization, as a result, decreasing inflammation in epididymal WAT (24, 99). The strategies aimed to maintain gut microbiota balance, reduce LPS, or block TLR4 signaling, may be useful in reducing adipose tissue inflammation and insulin-resistance in obesity.

It has been aware that interactions between host genetics, diet, and gut microbiota contribute to the development of obesity and metabolic syndrome (25, 26). Ussar and colleagues found that depending on the different genetic backgrounds of the mouse strains, HFD and other environmental factors might produce different changes in gut microbiota, had different impacts on adipose tissue inflammation, and created different interactions between the microbiome and the mouse models, as a result, leading to different susceptibility to obesity, hepatosteatosis, insulin resistance and other components of metabolic syndrome (100).

## 3 Factors Mediate the Cross-Talk Between Gut Microbiota and Adipose Tissues

It is well aware that the human gut microbiota composition can be influenced by gender, age, ethnicity, and geography (20, 21). Recent studies suggest that, although gut microbiota has a profound influence on the homeostasis of adipose tissues, the latter in turn can also affect the composition of gut microbiota *via* metabolites and cytokines.

### 3.1 Adipokines

Adipose tissues not only serve as energy storage but also as important endocrine organs capable of secreting lots of biologically active compounds that modulate whole body energy balance. The adipose tissues secrete bioactive peptides, referred to as adipokines, including leptin, adiponectin, retinol-binding protein 4 (RBP4), bone morphogenetic protein (BMP)-4, BMP-7, dipeptidyl peptidase 4 (DPP-4), vaspin, apelin, and progranulin (101). At a systemic level, adipokines control multiple biological processes in target organs, including adipose tissues liver, muscle, vasculature, heart, brain and pancreas, immune system, and many others (101). Several studies have proposed that the gut microbiota is a critical environmental factor in the modulation of the adipokine profiles.

#### 3.1.1 Leptin

Leptin plays an important role in the regulation of food intake, satiety, appetite, and energy expenditure (102, 103). Studies have shown that microbiota transplantation to GF mice increases fat mass with a higher production of leptin level, and this increase in leptin is proportional to the increase in body fat (104). Yao et al. found that leptin expression in GF mice increases with more CpG sites hypermethylated at the leptin promoter compared with conventional mice (103). High circulatory leptin could induce leptin resistance. Erik et al. found compared with GF mice, conventionally raised mice have reduced leptin sensitivity, as leptin treatment causes much less reduction in body weight and lower expression of the orexigenic peptides neuropeptide-Y (Npy) and agouti-related protein (Agrp). And leptin treatment increased leptin resistance-associated suppressors of cytokine signaling 3 (Socs-3) in the hypothalamus in conventional mice (105). These results indicate that the gut microbiota-associated increase of Socs3 might lead to leptin resistance and obesity in the conventional mice (105). Everard and colleagues have reported that prebiotic treatment is able to restore leptin sensitivity and improve metabolic parameters in HFD induced obese mice (106). Similar to those observations, prebiotic feeding decreases adiposity and increases plasma GLP-1 levels s in the ob/ob mice (106). Recently a randomized clinical trial suggested 12 weeks of probiotic administration significantly improved metabolic phenotypes and decreased leptin level compared with baseline values in obese women (107). However, whether and how gut microbiota mediate changes of leptin sensitivity/resistance in health and obesity are still unclear.

On the other hand, leptin may also have an impact on the gut microbiota. A daily supplement of leptin causes a higher proportion of *Clostridium genus and* a lower relative abundance of *Sutterella*, and enhances the expressions of interferon-α (TNF-α), mucin (MUC-2, MUC-3) during suckling period of rats, indicating a modulator role of leptin on the intestinal activation (108).

#### 3.1.2 Adiponectin

As another abundant adipokine, adiponectin also has a bilateral relationship with gut microbiota. Li and his colleagues have found that changes in gut microbiota are closely associated with increased plasma adiponectin levels, adiponectin-receptor-gene (AdipoR1 and AdipoR2), activated AMPK signal, and upregulated anti-obesity effects (109). Yao et al. found that alteration of the gut microbiota *via* antibiotics not only inhibits body weight gain, increases the expression of lipid oxidation and thermogenesis genes (including Pparα, Pgc-1α, Atgl) in adipose tissues, but also increases mRNA level of adiponectin (110). In addition, antibiotics reduces DNA methylation fractions of adiponectin promoters and down-regulate the level of DNA methyltransferase 1 (DNMT1) and 3a(DNMT3a) in HFD mice (110). Also, Membrez et al. found that microbiota depletion with norfloxacin and ampicillin could induce a higher circulating level of adiponectin while a lower level of LPS (111).

Adiponectin can influence the species richness of gut microbiota. Animals with daily adiponectin supplementation led to a lower relative abundance of *Proteobacteria phylum*, *Blautia* and *Roseburia*genus, and a higher proportion of the *Enterococcus* genus. Exogenous administration of adiponectin can increase the percentage of Tc TCRαβ+ and NKT cells and decrease the NK cells in intraepithelial lymphocytes (IEL) (108). Additionally, suckling period supplementation with adiponectin alters gut microbiota and enhances functions of the immune system (108).

#### 3.1.3 Fibroblast Growth Factor 21 (FGF21)

FGF21 is a member of the FGF superfamily and an important metabolic regulator, which is produced by adipose tissue, liver, and skeletal muscle (112, 113). Liver is considered as the major source of the endocrine FGF21 that circulates in the blood and regulates energy expenditure and insulin sensitivity (114). Recent studies indicated that JNK in adipose tissues could regulate adiponectin and hepatic FGF21 expression thus promoting a feed-forward regulatory loop to increase the crosstalk in different organs (115). Martin et al. discovered that gut microbiota mediates the hepatic FGF21 response to protein-restriction. During long-term dietary protein-restriction, the gut microbiota undergoes metabolic adaptations that stimulate hepatic FGF21 adaptive metabolic pathways (113). In the absence of gut microbiota, FGF21 is de-sensitized to the effects of protein-restriction (116). Additionally, changes in gut microbiome increase hepatic FGF21 expression and circulatory levels, which might be triggered by phosphorylation activation of eIF2αvia GCN2 (116, 117).

Ketogenic diet (KD) or carbohydrate-restricted diet can shift the gut microbiota toward “favorable bacteria” (i.e., *A. muciniphila, Lactobacillus*) and regulate lipid metabolism through PPARα/FGF21 pathway (117). Though short time KD enhances hepatic fatty oxidation and gluconeogenesis, long-term KD impairs FGF21 signaling, leading to glucose intolerance, insulin resistance, and macrophage infiltration, and steatosis in the liver (117, 118). A short time of carbohydrate-restricted diet consumption has been considered as a healthier lifestyle, while the underline mechanisms and debatable efficacy of this diet need more studies (22).

#### 3.1.4 Apelin

Apelin, a 36 amino-acid peptide, is the endogenous ligand of the G protein-coupled receptor APLNR. As one of the key hormones produced by adipose tissues, it has been demonstrated to modulate glucose homeostasis *via* AMPK and nitric oxide (NO)-dependent mechanisms (99). It contributes to regulate whole body well-being, such as blood pressure, cardiovascular and fluid homeostasis, lipid metabolism, food consumption, cellular proliferation, and angiogenesis (119). Studies have found that, compared with lean subjects, plasma apelin concentrations increase in obese subjects and in hyper-insulinemic obese mice (120). In type 2 diabetic mice, altered gut microbiota composition, e.g., higher abundance of *Firmicutes*, *Proteobacteria*, and *Fibrobacteres* phyla, regulates expression of apelin/APJ in adipose tissue through low-grade inflammatory tone and eCB system (121), indicating definite relationships between the gut microbiota and the apelin system. While the distinct roles of different bacteria in the development of diabetes are yet to be determined.

Taken together these findings suggest a close relationship between gut microbiota and the regulation of the adipokines in both physiological and pathophysiological conditions. However, the mechanisms behind these relationships require further studies.

### 3.2 Lipopolysaccharide

Gut microbiota-derived LPS has been defined as one of the key factors involved in the initiation and development of inflammation and obesity related metabolic disorders (122). In response to HFD feeding, the production and transportation of LPS from the gut lumen toward target tissues can induce increased plasma LPS levels, resulting in metabolic endotoxemia and low-grade inflammation. Specifically, HFD causes gut microbiota-dependent impairment of the tight junction proteins, such as occludin, occludens-1, and zonula, which are important for the gut barrier function. The antibiotic treatment abolishes diet-induced gut permeability, diminishes endotoxemia absorption, and lowers inflammation and glucose intolerance (123, 124). It has been established that, compared with controls, obese individuals have higher plasma levels of LPS, which were closely associated with intra-abdominal adipose tissue mass but only moderately correlated with subcutaneous fat mass (125). Consistently, both plasma LPS levels and the LPS-containing microbiota in the gut were dramatically induced by a 4-week HFD feeding. Continuous subcutaneous infusion of LPS for 4 weeks in mice presented a similar phenotype in HFD fed mice and increased the expression of inflammation markers in adipose tissues by the LPS/CD14/TLR4 system or TLR4/MD-2 complex (76, 81). Hersoug et al. has proposed that scavenger receptor class B type 1 (SR-BI) binds to both lipids and LPS and stimulates LPS transfer to other lipoproteins with the help of translocases. LPS can induce transcytosis of lipoproteins over the endothelial barrier and endocytosis in adipocytes by felicitating the SR-BI binding (126). Large sizes of adipocytes with higher metabolic activity absorb more LPS-rich lipoproteins, which may contribute to the M2 to M1macrophages polarization, and as a result, increase LPS delivery into the hypertrophic adipose tissue (126).

### 3.3 Short-Chain Fatty Acids

SCFA, including acetate, butyrate, and propionate (which make up more than 95% of the SCFA content), are extracted from gut microbial fermentation of foods that cannot be digested due to the lack of appropriate enzymes (127). SCFA can be largely absorbed by colonic epithelium, used as energy resources, and play a vital role in the maintenance of gut health and metabolism homeostasis.

SCFA produced through fiber fermentation in the colonic region is known to regulate glucose and lipid metabolism. On the one hand, SCFA might increase intestinal energy harvesting capabilities and induce obesity. Fecal SCFA concentration has been reported to increase in ob/ob mice or obese humans. In ob/ob mice, increased *Firmicutes* to *Bacteroidetes* ratio is connected to increased caecal concentrations of acetate and butyrate or acetate and propionate respectively (128). In HFD fed rats, by using ^13^C-acetate labeled method to measure tissue acetate concentrations, Perry and his colleagues found that increased production of acetate by gut microbiota could activate parasympathetic nervous system and increase glucose-stimulated insulin secretion, hyperphagia, and obesity (129). Individuals with obesity have a higher concentration of SCFA in fecal compared with lean controls, who have significantly higher fecal concentrations of the genus *Bacteroides*but and lower *Ruminococcus flavefaciens* and *Bifidobacterium* (130), suggesting that SCFA metabolism might act as an essential factor in obesity.

However, on the other hand, studies have also found that SCFA increases energy expenditure and can reduce or reverse body weight gain and adiposity (131, 132). Lu et al. has demonstrated that supplementation of SCFA admixtures could change the composition of gut microbiota and reduce body weight by enhancing triglyceride hydrolysis and FFA oxidation in the adipose tissue, stimulating mitochondrial biogenesis and beige adipogenesis, and suppressing chronic inflammation (132). Oral supplementation of sodium butyrate leads to body weight loss and improves fasting blood glucose and insulin tolerance of obese mice by increasing energy expenditure and fat oxidation (133). Butyrate administration increases mitochondrial function and biogenesis and elevates AMPK and p38 activities *via* a PPARγ-dependent pathway in the BAT (133, 134). Furthermore, supplementation of acetate can result in an increase in GLP-1 and PYY, appetite suppression, and hypothalamic neuronal activation patterning, thereby reducing body weight, food intake, and fat mass (135).

SCFA can directly act on adipose tissues *via* G protein coupled receptor 41 and 43 (GPR41 and GPR43) dependent manner. By inhibiting intracellular lipolysis (mainly through acetate and propionate) and increasing the lipid buffering capacity of adipose tissues (mainly by propionate), SCFA can lead to a reduction of lipid overflow and decrease of ectopic fat accumulation, thereby positively regulating insulin sensitivity (132, 136). SCFA might be also involved in adipogenesis *via* GPR43 in rodent adipocytes. Hong et al. found expression of GPR43 was up-regulated in adipose tissues of HFD mice and also in differentiated 3T3-L1 adipocytes (137, 138). And both propionate and acetate can elevate GPR43 expression with up-regulation of PPAR-γ2 and inhibit lipolysis in a dose-dependent manner (138).

SCFA might also mediate cold exposure induced adipose tissue thermogenesis. Cold exposure alters SCFA production while some SCFA including acetate and propionate were decreased in mice after antibiotics (ABX) treatment, however, butyrate was not altered and, under cold exposure, *Lachnospiraceae*, which belongs to butyrate producing bacteria *Clostridia*, was increased (53). Butyrate sodium administration to antibiotics treat mice can increase their body temperature and Ucp1 expression in BAT and subcutaneous WAT. Using the isotope labeling method to trace^12^C/^13^C butyrate, it has been reported that butyrate functions through the gut-brain axis instead of having a direct effect on adipose tissues (53, 63). Butyrate could reduce appetite and activate BAT *via* suppressing the activity of orexigenic neurons and the expression of NPY (139). Cold microbiota transplantation can also result in significant changes in SCFA, increasing the concentration of acetic acid, butyric acid, isobutyric acid, and isovaleric acid. Besides, cold microbiota transplantation can activate the β3-adrenoceptor-cAMP-PKA signaling pathway (55), indicating that gut microbiota can activate the NE system *in vivo*, probably through microbiota metabolite SCFA.

### 3.4 Bile Acids

Secreted in the liver from cholesterol, bile acids are metabolized in the intestine through the gut microbiota. Many researchers have proven that gut microbiota can regulate the composition of bile acids. In GF mice, the bile acid pool is primarily comprised of conjugated bile acids (140). The bile acids function *via* the nuclear Farnesoid X receptor (FXR) and the G protein-coupled membrane receptor 5 (GTR5), which are involved in numerous metabolic pathways in the host (140, 141).

Past research that has used hepatic and intestinal disruption of the FXR have demonstrated that the liver and the intestine both acts as central organs for FXR signaling-dependent glucose and lipid control. FXR deficiency can reduce body weight gain and adipose tissue mass, and improve glucose homeostasis and insulin sensitivity in HFD mice (142). Specific intestinal FXR activation protected against the development of obesity through increased thermogenesis and WAT browning (142, 143). Another bile acid-responsive receptor is TGR5, or, the G protein bile acid activated receptor (GPBAR)-1, regulates glucose homeostasis by bolstering energy expenditure in BAT and in muscle and enhancing GLP-1 release in intestinal L cells (144).

Cold exposure can change the composition of bile acid pool by inducing hepatic enzymes involved in the conversion of cholesterol to primary bile acids (49), which, in turn, may mediate the action of cold exposure on the shape of the gut microbiome and adaptive thermogenesis promotion. Genetic and pharmacological interventions that targeted CYP7B1 (cytochrome P450, family 7, subfamily b, polypeptide 1), the key enzyme of synthesis and biliary excretion of bile acids, have hindered the rise in fecal bile acid excretion, altered the bacterial composition of the gut and, more importantly, regulated thermogenic responses, implying that bile acids may play a role as crucial metabolic effectors under sustained BAT activation (50).

As mentioned above, the diet was closely associated with gut microbiota and bile acids pool (64, 142). Wang et al. found that HFD significantly increased the deoxycholic acid (DCA) content in faces and administration of DCA promoted M1 macrophage polarization *via* NF-κB/ERK/JNK signaling (145). Li and colleagues have reported that compared with normal diet, CR decreased the conjugated non-12α-hydroxylated (12OH) BAs, such as tauro-β-muricholic acid (TβMCA), tauro-ω-muricholic acid (TωMCA), tauroursodeoxycholic acid (TUDCA), and taurochenodeoxycholic acid (TCDCA) (64). Interestingly, the gut microbiota will shift towards favoring bacteria that can harvest energy more efficiently during CR. When coming back to a normal diet or high-fat diet, gut microbiota will be reshaped, and the abundance of *parabacteroides distasonis* and 12OH BAs for efficient fat adsorption increase significantly, leading to overweight again (64), suggesting that BAs are important mediators for gut microbiota regulated body weight.

### 3.5 Endocannabinoid System

The eCB system is comprised of endogenous lipids that trigger specific G protein-coupled receptors termed cannabinoid receptors 1 and 2 (CB1 and CB2). It has been demonstrated that the eCB system consists of more than 100 lipid mediators and 50 proteins, such as CB1, CB2, *N*-arachidonoyl-ethanolamine (AEA), 2-arachidonoyi-glycerol (2-AG), and the anabolic and catabolic enzymes for the endocannabinoids (146, 147). The eCB system has the ability to regulate gut-barrier function, gut permeability, and metabolic endotoxemia in obesity, and control energy balance by manipulating the metabolic process, energy intake and fat accumulation in adipocytes (148–150). The eCB also boosts fat storage in adipocytes by inducing adipogenesis and triacylglyceride production. The CB1 inhibits the activity of AMPK and reduces the AMPK-associated lipolysis (148). Geruts et al. has demonstrated that db/db mice displayed increased eCB signals in adipose tissues, and apelin and APJ expression were decreased by the eCB system under normal conditions (121). The activation of its specific receptor CB1R in mice stimulates the expression of PPARγ and LPL in WAT and increases the expression of genes involved in adipocyte differentiation, including PPARγ, C/EBPα, AP2, and genes involved in lipogenesis, such as SREBP-1c, ACC, FAS markedly (150, 151).

Chronic CB1 antagonism promotes body weight loss in diet-induced obese mice by increasing energy expenditure insulin-stimulated glucose application and significant stimulation of BAT activation (152). Paola et al. found chronic *N*-oleoyl-ethanolamine (OEA) administration to mice in normal diet could shift *Firmicutes/Bacteroides* ratio, decrease *Lactobacillus*, and reduce intestinal cytokines expression (153). In obese humans, supplementation of OEA decreased BMI and waist circumference, which might due to the activation of PPARα (147). And the administration of palmitoyl ethanolmaide (PEA), which is a member of *N*-acyl ethanolamide (NAE) similar to AEA, could decrease body weight, food intake, and fat mass in HFD rats (154). So the design and development of eCB system agonists, antagonists, and allosteric modulators may represent a therapeutic way for gut dysbiosis and related metabolism diseases. However, the mechanism behind this is largely unclear.

## 4 Conclusion Remarks and Future Perspective

**Figure 1:**
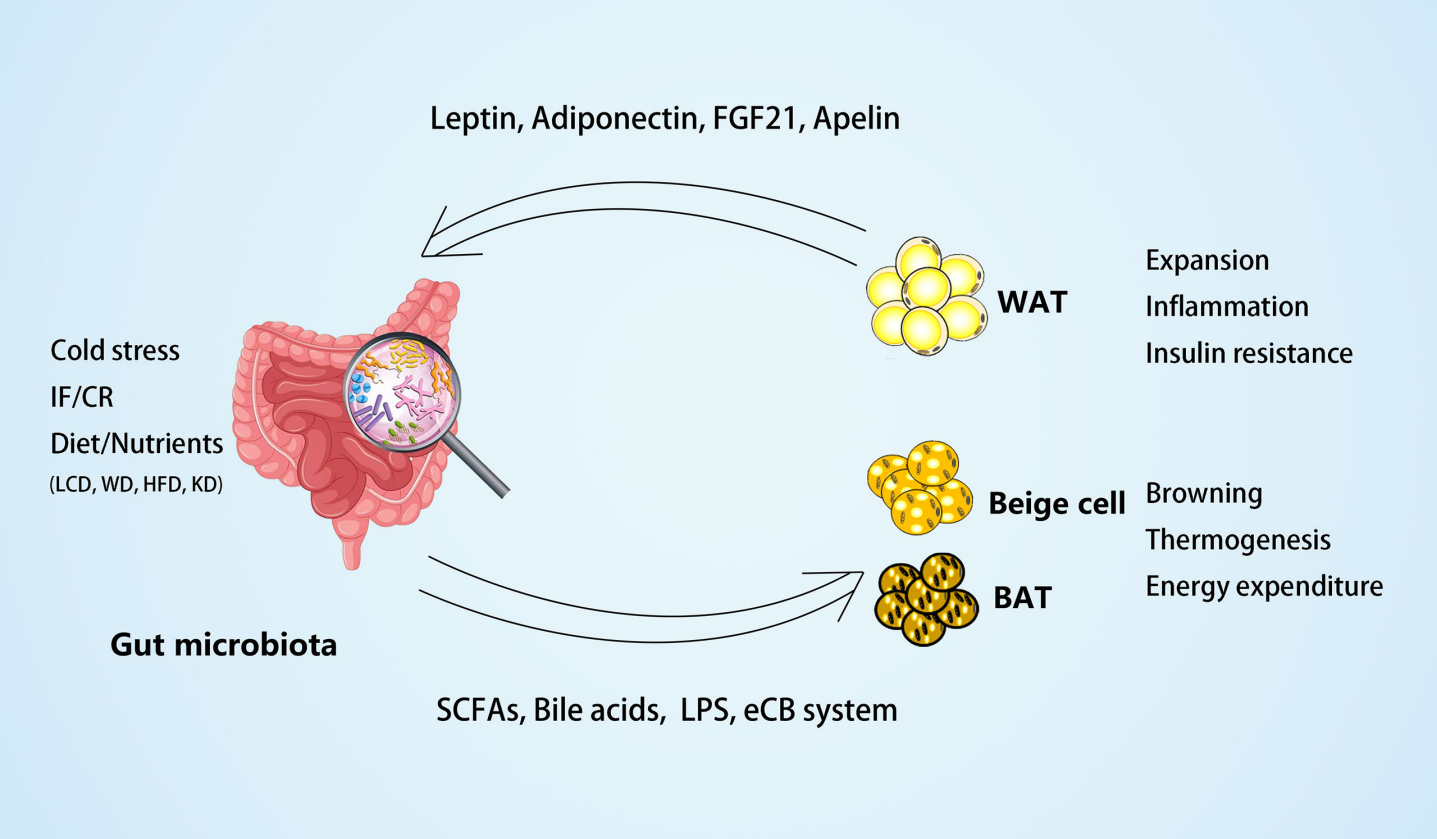
The crosstalk between gut microbiota and adipose tissues. On the one hand, the gut microbiota and microbial-derived metabolites may mediate the external factors, such as dietary foods, to regulate white adipose tissue (WAT) expansion, inflammation, and insulin resistance in obesity. Different diets may have different effects on the gut microbiota, such as low carbohydrate diet (LCD) (22) can increase *Bacteroidaceae Bacteroides* and reduce body weight effectively; whereas western diet (WD) (24) or high-fat diet (HFD) (43, 76) can increase dysbiosis of gut microbiota and inflammation of adipose tissues; and ketogenic diet (KD) (117) can increase *A. muciniphila, Lactobacillus* and regulate lipid metabolism through PPARα/FGF21 pathway. On the other hand, the gut microbiota and its metabolites may mediate environmental temperature, intermittent fasting (IF), or caloric restriction (CR) in the process of energy expenditure by promoting browning and thermogenesis of beige and brown adipose tissue (BAT). In the meantime, some adipokines, including leptin, adiponectin, FGF21, and apelin, produced by adipose tissues can act on the gut microbiota to modulate its composition.

Recent research has indicated that the gut microbiota can contribute to host metabolism by cross-talk with adipose tissues. On the one hand, the gut microbiota modulates adipogenesis and energy expenditure and alleviates obesity by promoting the browning of WAT and the activity of BAT. On the other hand, microbial-derived metabolites, such as SCFA and LPS, may act as the central factors in the pathogenesis of adipose inflammation, insulin resistance, and obesity. Additionally, the gut microbiota is a crucial environmental factor in adipokine profile regulation, which, in turn, may mediate the functional modulation of adipose tissues to the microbiota (**Figure 1**).

Although a number of inconsistent findings have been reported, perhaps as a result of differences in mice strain, housing conditions, or diet, current studies have shown changes of gut microbiota composition, specific metabolites and some exogenous substances have impacts on modulating WAT browning, BAT activity, and thermogenesis. However, the mechanisms underlying the relationship between gut microbiota changes and browning of WAT or BAT activity were still unclear and, more importantly, evidence collected from studies in humans is needed to be verified. In addition, despite these associations, the causal pathways and the mechanisms underlying the relations between gut microbiota and adipose tissues in inflammation and risks of obesity associated diseases have yet to be fully classified.

Further characterization of the stimulators of a steady, low-grade, inflammatory response in people with obesity might provide different evidence for novel intervention methods to help to alleviate the risk of obesity and related disorders. These changes would help to guide the clinical application of LCD to overweight and obese patients (22). Additionally, due to their beneficial effects on the host, some probiotic bacteria have been widely used as a health care product. For example, *Bifidobacterium* genera has been added to many foods and medicines to regulate gut microbiota and metabolism. Some others, such as *Lactobacillus paracasei* (42, 44). *Akkermansia muciniphila* (63), *Faecalibacterium prausnitziiare, Parabacteroides distasonis* and *Clostridium leptum* (94–97) have been reported to improve insulin sensitivity and alleviate the inflammation of adipocytes. Also, some microbial-derived metabolites such as certain SCFA and bile acids, may provide enlightenments to treat obesity. And after 3-month treatment of berberine (BBR), the baseline of *Alistipes* and *Blautia* changed that was closely associated with altered serum lipids levels (155). And metformin treatment significantly altered microbiota composition and its metabolites, including decreased *Roseburia faecis* and *Roseburia intestinalis* and increased butyrate, acetate and valerate (156). These studies implied that monitoring gut microbiota might be a potential way to predict the pharmacotherapeutic efficacy of some medicine. In the future, targeting the gut microbiota and their metabolites may provide a promising approach for therapeutic interventions and treatment of obesity and related metabolic diseases.

## Author Contributions

DW performed the literature search and prepared the original draft, HW & LX performed the literature search and edited the manuscript, and FH had the idea for the article and critically revised the work. All authors read and approved the final manuscript.

## Funding

This work was supported by grants from National Nature Science Foundation of China (91957113& 31871180), National Key R&D Program of China (2020YFA0803604), and Natural Science Foundation of Hunan Province (2019JJ40410) to F.H.

## Conflict of Interest

The authors declare that the research was conducted in the absence of any commercial or financial relationships that could be construed as a potential conflict of interest.

## Publisher’s Note

All claims expressed in this article are solely those of the authors and do not necessarily represent those of their affiliated organizations, or those of the publisher, the editors and the reviewers. Any product that may be evaluated in this article, or claim that may be made by its manufacturer, is not guaranteed or endorsed by the publisher.
